# Using animal models to determine the significance of complement activation in Alzheimer's disease

**DOI:** 10.1186/1742-2094-1-18

**Published:** 2004-10-12

**Authors:** David A Loeffler

**Affiliations:** 1 Department of Neurology, William Beaumont Hospital Research Institute, Royal Oak, MI 48073, USA

**Keywords:** Alzheimer's disease, animal models, complement activation, transgenic mice

## Abstract

Complement inflammation is a major inflammatory mechanism whose function is to promote the removal of microorganisms and the processing of immune complexes. Numerous studies have provided evidence for an increase in this process in areas of pathology in the Alzheimer's disease (AD) brain. Because complement activation proteins have been demonstrated *in vitro *to exert both neuroprotective and neurotoxic effects, the significance of this process in the development and progression of AD is unclear. Studies in animal models of AD, in which brain complement activation can be experimentally altered, should be of value for clarifying this issue. However, surprisingly little is known about complement activation in the transgenic animal models that are popular for studying this disorder. An optimal animal model for studying the significance of complement activation on Alzheimer's – related neuropathology should have complete complement activation associated with senile plaques, neurofibrillary tangles (if present), and dystrophic neurites. Other desirable features include both classical and alternative pathway activation, increased neuronal synthesis of native complement proteins, and evidence for an increase in complement activation prior to the development of extensive pathology. In order to determine the suitability of different animal models for studying the role of complement activation in AD, the extent of complement activation and its association with neuropathology in these models must be understood.

## Background

### Alzheimer's disease and complement activation

A variety of inflammatory processes are increased in regions of pathology in the Alzheimer's disease (AD) brain [1-4]. There is a reciprocal relationship between this local inflammation and senile plaques (SPs) and neurofibrillary tangles (NFTs); both SPs and NFTs, as well as damaged neurons and neurites, stimulate inflammatory responses [5], and inflammatory processes exert multiple effects, some of which promote neuropathology [6-8]. Numerous retrospective studies have shown that long-term administration of nonsteroidal anti-inflammatory drugs (NSAIDs) to individuals with arthritis significantly reduces the risk for these individuals for developing AD [9]. These findings, together with the demonstration of elevated glial cell activation [10-12], complement activation [13-15], and increased acute phase reactant production [16-19] at sites of pathology in the AD brain, support the hypothesis that local inflammation may contribute to the development of this disorder [20]. Although a short-term trial of AD patients with the NSAID indomethacin suggested protection from cognitive decline [21], subsequent trials with other anti-inflammatory drugs have found no evidence for slowing of the dementing process [22-25]. These findings underscore the current perception of CNS inflammation as a "double edged sword" [26,27], with neuroprotective roles for some inflammatory components and neurotoxic effects for others [28-30].

**Figure 1:**
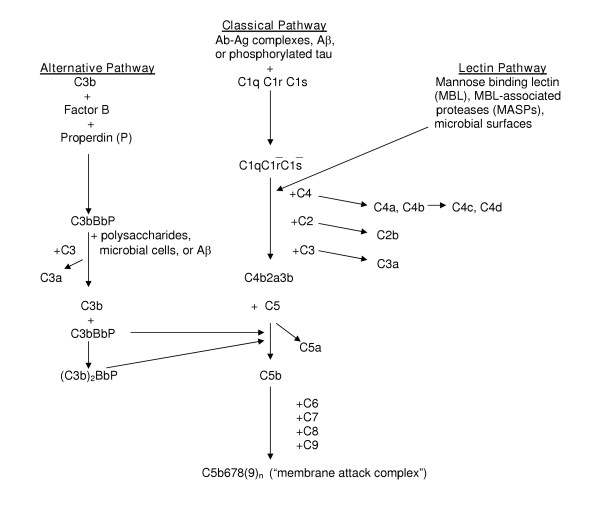
Schematic diagram of classical, alternative, and lectin complement activation pathways. There is evidence for activation of the classical and alternative pathways in the AD brain. (Adapted from Sahu and Lambris, 2000 [58]).

The significance of complement activation, a major inflammatory mechanism, in AD is particularly problematic. The complement system is composed of more than 30 plasma and membrane-associated proteins which function as an inflammatory cascade. Complement activation promotes the removal of microorganisms and the processing of immune complexes. The liver is the main source of these proteins in peripheral blood, but they are also synthesized in other organs including the brain [31]. Protein fragments generated during activation of the system enzymatically cleave the next protein in the sequence, generating a variety of "activation proteins" with diverse activities (Table 1). Three complement pathways, the classical, alternative, and lectin-mediated cascades, have been identified (Fig. 1). Full activation results in the generation of C5b-9, the "membrane attack complex" (MAC), which penetrates the surface membrane of susceptible cells on which it is deposited and may result in cell death if present in sufficient concentration. The presence of early complement activation proteins [32-37] and of the MAC [38-42] has been demonstrated by immunocytochemical staining in the AD brain. Subsequent studies found that complement activation increases Aβ aggregation [43,44] and potentiates its neurotoxicity [45], attracts microglia [46,47], promotes microglial and macrophage secretion of inflammatory cytokines [48,49], and induces neuronal injury, and sometimes neuronal death, via the MAC [50]. These findings suggested that complement activation might contribute to the neurodegenerative process in AD. However, recent studies have also revealed neuroprotective functions for some complement activation proteins, including *in vitro *protection against excitotoxicity [51,52] and Aβ-induced neurotoxicity [53], as well as anti-apoptotic effects [54,55]. Further, C1q, the first complement protein to be deposited on cell membranes during activation of the classical complement sequence, may facilitate the clearance of Aβ by microglia [56], although this is controversial [57]. Understanding the role of complement activation in AD is of clinical relevance because some complement-inhibiting drugs are available, and others are being developed (see reviews by Sahu and Lambris [58], and Morgan and Harris [59]). Conditions for which these agents are currently being investigated include stroke [60], organ transplantation [61], glomerulonephritis [62], ischemic cardiomyopathy [63], and hereditary angioedema [64]. Modulation of CNS complement activation in experimental animal models of AD, both by treatment with complement-inhibiting drugs and by generation of AD-type pathology in complement-deficient animals, should be useful for obtaining a greater understanding of the role of this process in the development of AD-type pathology. Unfortunately, knowledge of the extent of complement activation in animal models is lacking. This paper will review (a) criteria for an optimal animal model to study this issue, (b) present knowledge about complement activation in animal models of AD, and (c) additional animal models which offer alternatives for addressing this question.

**Table 1:** Biological activities of complement activation proteins, with relevance to AD.

Name	Biological activity
C1q	Enhances Aβ aggregation [43,44]; may facilitate Aβ clearance [56]; enhances Aβ-induced cytokine secretion by microglia [49]
C3a	Anaphylatoxin (increases capillary permeability) [155] ; protects neurons vs. excitotoxicity [52]
C3b	Immune adherence and opsonization [89] (may facilitate Aβ clearance by phagocytic microglia)
C4a	Anaphylatoxin (weak) [156]
C5a	Anaphylatoxin; protects neurons vs. excitotoxicity [51]; chemotaxic attraction of microglia [46,47]; inhibits apoptosis 54; increases cytokine release from Aβ-primed monocytes [48]
C5b-9	Neurotoxicity [50]; sublytic concentrations may have both pro- and anti- inflammatory activities [157]

### Criteria for an optimal animal model for studying AD-related complement activation

While animal models of human disease generally have similar pathological findings to the human disorders, distinct differences remain. These models may be appropriate for studying some aspects of a disease process, while less suitable for others. To determine the significance of complement activation in the development of AD-type pathology, for example, some animal models may be of value primarily for investigating the relationship between early complement activation and SP and NFT formation, whereas others may be more relevant for studying the role of the MAC in neuronal loss.

#### 1. Complete activation of complement

Investigators at the Academic Hospital Free University in Amsterdam first reported the presence of early activation proteins in the classical complement cascade in the AD brain [32-34,36,37]. The MAC was not detected. However, further studies by other laboratories convincingly demonstrated the MAC, by a variety of techniques, in AD specimens [38-42]. The Dutch group has more recently reported detection of the MAC in brain specimens from subjects with dementia with Lewy bodies who met CERAD neuropathological criteria for AD [65]. The MAC has similarly been reported in SPs from subjects with Down's syndrome [66] and with familial British dementia [67], disorders in which typical AD-type neuropathology is present. An optimal animal model for studying AD-related complement activation should therefore have complete complement activation.

#### 2. Association of complement activation proteins with neuropathology

Complement proteins are detectable on or closely associated with SPs, NFTs, and dystrophic neurites in the AD brain. These findings are in agreement with *in vitro *studies indicating that Aβ and tau protein, the major components in SPs and NFTs, can fully activate human complement [42,68-71]. Although the above studies suggested that complement is activated principally by the aggregated forms of Aβ and tau, soluble, non-fibrillar Aβ may also be capable of activating complement [72]. In contrast to the robust staining of complement proteins in mature plaques, immunoreactivity to these proteins in diffuse plaques has generally been below the level of detection, though it has been reported in some studies [36,73,74]. Complement activation in the AD brain is increased primarily in regions containing extensive pathology (e.g., the hippocampus and cortex), and whether early complement components are also present in the diffuse plaques that develop in the AD cerebellum is controversial [74,75]. The above findings suggest that complement activation in an optimal animal model of AD should be associated with SPs and, in those models in which neurofibrillary pathology occurs, with NFTs.

#### 3. Initiation of complement activation early in development of pathology

How the increased complement activation in AD relates to the development of SPs and NFTs, and to neuronal loss, is unclear. Immunocytochemical staining for complement activation proteins in the aged normal human brain is generally faint, and may be below the level of detection [42,69,73]; of relevance is a recent report describing extensive neuron-associated C1q reactivity in a cognitively normal subject with neuropathological findings limited to diffuse cortical plaques [76]. Elderly "high pathology controls," lacking dementia but with increased numbers of entorhinal NFTs and neocortical Aβ deposits, have a slight increase in the percentage of C5b-9-immunoreactive plaques in comparison with aged normal subjects, though this percentage is far lower than in the AD brain [39]. A recent study in our laboratory [77] used enzyme-linked immunosorbent assay (ELISA) to measure the concentrations of two early complement activation proteins, C4d and iC3b, in brain specimens from AD and normal subjects. ELISA is more sensitive than immunocytochemical staining, though it provides no information regarding the cellular association of complement immunoreactivity. Increased concentrations of these early complement activation proteins were present in some aged normal specimens. These reports suggest that early complement activation may increase prior to the development of plaques and NFTs. Similar findings are desirable in an optimal animal model for studying AD-related complement activation.

#### 4. Increased CNS production of native complement proteins

Both mRNA expression and protein synthesis of native complement proteins are increased in the AD brain [78-80]. (Note: the distinction between detection of native complement proteins, vs. detection of complement activation proteins, has frequently been blurred. In some studies in which immunoreactivity to complement activation proteins (C3c, C4c, C4d) has been reported, the antisera used were also capable of detecting the respective native complement proteins (C3 or C4) [40,80]. Only when antisera are used whose immunoreactivity is limited to activation-specific neo-epitopes can complement activation be confirmed. The paucity of antisera which can detect complement activation proteins in experimental animal models is a significant obstacle to determining the extent of complement activation in these models.) In addition to neurons, complement proteins are synthesized by other cells in the CNS including microglia, astrocytes, oligodendrocytes, and endothelial cells [31]. The biological effects of these activation proteins are mediated by numerous regulatory proteins including CD59, clusterin, vitronectin, C1-inhibitor, C4-binding protein, decay-activating factor, and Factor H, which inhibit different steps in the complement cascade. All of these regulatory proteins are produced in the human brain, but less is known about their CNS synthesis in other species [31]. The status of some of these regulatory proteins in AD is unclear; for example, there are conflicting reports regarding the up-regulation of C1-inhibitor [81,82] and CD59 [41,82,83]. Thus, while an optimal animal model for studying AD-related complement activation should have up-regulated CNS synthesis of complement proteins, the alterations that should be present in complement regulatory proteins are less clear.

#### 5. Alternative as well as classical complement activation

Complement activation in the AD brain was initially thought to be limited to the classical pathway, but recent reports have also indicated increased concentrations of the alternative activation factors Bb and Ba, and Factor H, a regulatory factor for the alternative pathway, in the AD brain [84,85]. Alternative complement activation has also been reported in other familial dementias with pathologies similar to AD [67]. Therefore, while activation of the classical pathway is an absolute requirement for an optimal animal model of AD-related complement activation, an increase in the alternative pathway is also desirable.

### Complement activation in animal models of AD: present knowledge

The examination of complement activation in experimental models of AD has been limited to mice and rats. The extent of complement activation and its relationship to the development of AD-type neuropathology have generally not been determined in these studies.

#### APP/sCrry mouse

Increased complement activation was induced by overproduction of transforming growth factor beta1 (TGF-β1) in transgenic mice expressing mutations in the human amyloid precursor protein (hAPP) gene. The APP mutations expressed in these mice have been associated with early-onset, familial AD [86]. The TGF-β1 overproduction resulted in a 50% reduction in Aβ accumulation in the hippocampus and cerebral cortex [87]. Because the production of soluble Aβ was unchanged, these results suggested that reduction in Aβ may have been due to its increased clearance by microglia. A subsequent study by the same investigators [88] found that the mRNA level of C3 in the cerebral cortex was 5-fold higher in APP/TGF-β1 mice than in APP mice at 2 months of age (prior to deposition of Aβ) and 2-fold higher at 12–15 months, when senile plaques are present. Thus, in this model, increased CNS synthesis of C3 precedes senile plaque formation. Because C3b, an activation protein produced by cleavage of C3, functions as an opsonin [89], the increased C3 levels together with the reduced Aβ deposition in the APP/TGF-β1 mice suggested a neuroprotective role for complement in this model. To investigate this possibility, the APP mice were crossed with mice expressing soluble complement receptor-related protein y (sCrry), a rodent-specific inhibitor of early complement activation [90]. APP/sCrry mice had a 2- to 3- fold increase in Aβ deposition in the neocortex and hippocampus at 10–12 months of age, together with a 50% loss of pyramidal neurons in hippocampal region CA3. The authors concluded that complement activation may protect against Aβ-induced toxicity, and may reduce the accumulation or promote the clearance of amyloid and degenerating neurons [88]. Neuroprotective functions (protection against excitotoxicity) have been demonstrated *in vitro *for C3a [52], and the increased neuronal loss in the APP/sCrry mouse may be due to decreased production of C3a as well as the opsonin, C3b. However, whether inhibition of complement activation in the AD brain would similarly result in increased neuropathology is unclear, because complement activation in AD is likely to be more extensive than in the APP mouse. Although no peer-reviewed articles have appeared in which the extent of complement activation in the APP mouse has been examined, two abstracts have dealt with this issue. Yu et al. [91] reported C3, C5, and C6 immunoreactivity to thioflavin-S-reactive plaques, whereas McGeer et al. [92] found only weak complement staining of plaques and slight upregulation of complement proteins. Significantly, neither study reported detection of the MAC. At least two factors, in addition to the lack of NFTs, mitigate against complement activation in the APP mouse being equivalent to that in AD: (a) the mouse complement system is functionally deficient, as mouse C4 lacks C5 convertase activity [93] and many mouse strains have low complement levels relative to other mammals [94], and (b) mouse C1q binds less efficiently to human Aβ than does human C1q, resulting in less activation of mouse complement than of human complement in the presence of human Aβ [95].

#### PS/APP mouse

In addition to APP, mutations in the gene encoding for presenilin-1 (PS-1) have also been associated with familial AD [96]. The PS/APP mouse carries both of these transgenes and has been extensively used as a model for studying processes relating to the formation of SPs. Aβ deposition occurs more rapidly in these mice than in the single transgenic APP mouse [97]. In neither model does NFT formation occur. Aβ deposition in PS/APP mice is initially detected at 3 months of age, and increases with age; total Aβ burden peaks at one year of age, although the percentage of Aβ that is fibrillar (thioflavin-S reactive) increases up to 2 years of age. Matsuoka et al. [98] described the CNS inflammatory response to Aβ in these animals. Activated astrocytes and microglia increased in parallel with total Aβ and were closely associated with both diffuse and fibrillar plaques. C1q immunoreactivity was detected at both 7 and 12 months of age, co-localizing with activated microglia and fibrillar Aβ. These findings were similar to those in the AD brain in that complement activation was associated with SP formation. The extent of complement activation was not addressed in this study.

#### APP (Tg2576)/C1q-deficient mouse

Fonseca et al. [99] investigated the role of C1q in AD by crossing Tg2576 (APP) mice [100] and APP/PS1 mice with C1q knockout mice [101]. C1q immunoreactivity was associated with plaque formation in the APP Tg2576 animals, as previously reported by Matsuoka et al. [98]. In both the Tg2576/C1q^- ^and APP/PS1/C1q^- ^animals, lack of C1q did not alter either plaque density or the time course of plaque deposition. Neuronal cell numbers (NeuN^+ ^cells), assessed only in the Tg2576 (APP) mouse, were not changed by the absence of C1q; however, immunoreactivity to MAP-2 (a marker for neuronal dendrites and cell bodies) and synaptophysin (a marker for presynaptic terminals) in the hippocampus (region CA3) was increased 2-fold in the APP/C1q^- ^animals, compared with APP mice. Microglial and astrocytic activation was significantly reduced in the APP/C1q^- ^animals. These results were interpreted to suggest that in these animal models of AD, (1) early complement activation (as indicated by C1q deposition) in response to fibrillar Aβ deposition might be responsible for the chemotactic attraction of activated glial cells, and (2) the activated microglia, while unable to clear fibrillar Aβ, may have contributed to the loss of neuronal integrity indicated by reduced MAP-2 and synaptophysin staining in the APP mice. By recruiting activated microglia, complement activation could potentially contribute to neuronal injury even if full activation (MAC formation) does not occur.

#### Postischemic hyperthermic rat model

Coimbra and colleagues [102] described progressive neuronal loss in the hippocampus and cerebral cortex in rats subjected to common carotid artery occlusion to produce transient forebrain ischemia, as an animal model for stroke. The post-surgical hyperthermia which occurs spontaneously in these animals was suggested to promote the infiltration of microglia, whose secretory products increased the subsequent neuronal loss. A later study by the same group [103] found that subjecting the rats to post-surgical hyperthermia (38.5 – 40°C) increased microglial and astrocytic infiltration and accompanying neuronal loss, and resulted in the formation of AD-type pathology. Aβ-reactive diffuse plaques were detected in the cerebral cortex at 2 months post-surgery, with more compact plaques in the hippocampus and cortex by 6 months. Increased ubiquitin and phosphorylated tau immunoreactivity was observed at both time points, together with staining for C5b-9 in the somatosensory cortex. The MAC immunoreactivity co-localized with acid fuchsin staining, a marker for neuronal death [104]. Other complement proteins were not evaluated in these studies. This is apparently the only animal model of AD in which full complement activation has been reported. It is noteworthy that while both SPs and neurofibrillary pathology were present in these animals, the MAC apparently did not co-localize with these structures, unlike in AD.

#### Acute lesioning

Alterations in native complement mRNA and protein levels have been evaluated in the rat hippocampus following experimental induction of acute neuronal injury. These surgical and pharmacological procedures result in neuronal loss in the entorhinal cortex, and deafferentation of hippocampal neurons, similar to that which occurs in AD [105]. Selective damage to the rat hippocampus has been induced by surgical transection of the perforant pathway, which runs between the entorhinal cortex and the molecular layer of the dentate gyrus [106,107], systemic administration of the excitotoxin kainic acid [108,109], or injection of the neurotoxin colchicine into the dorsal hippocampus [109]. Surgical transection of the perforant pathway increased C1qB mRNA in the entorhinal cortex and hippocampus [106] and C9 immunoreactivity in the hippocampus [107]. Injection of kainic acid similarly increased C1qB and C4 mRNA expression and C1q immunoreactivity in the hippocampus [108,109]. Colchicine infusion into the dorsal hippocampus, which selectively damages granule cells of the dentate gyrus, produced elevated mRNA expression of hippocampal C1qB and C4 [109]. Though the acute neuronal damage in these studies differs from the chronic, progressive neurodegenerative process that occurs in AD, these results demonstrated that the neuronal response to injury includes upregulation of native complement protein synthesis. The significance of this upregulation, i.e. whether it promotes neuroprotection or neurotoxicity, was not addressed.

#### Infusion of Aβ and C1q into rats

Frautschy et al. [56] examined the effects of infusion of human C1q and oral administration of rosmarinic acid on glial cell proliferation (microgliosis and astrocytosis), plaque load, and memory (Morris water maze) in Aβ-infused rats. Rosmarinic acid inhibits both the classical and the alternative complement cascades, by covalent binding to newly formed C3b [110]; it also possesses anti-inflammatory [111,112], anti-oxidative [113], and anti-amyloidogenic properties [114]. Gliosis was greater with C1q and Aβ infusion than with Aβ alone. Plaque density was decreased by C1q infusion (note: this result differs from the *in vitro *study of Webster et al. [57], in which C1q was found to inhibit microglial phagocytosis of Aβ, and also from the recent study of Fonseca et al. [99] in which C1q deficiency had no effect on plaque density in APP mice), but, curiously, performance in the water maze worsened. Treatment with rosmarinic acid had the opposite effect; though plaque load increased, memory was improved. These findings were interpreted as suggesting that C1q and/or complement activation may, by promoting microglial activation, worsen memory independent of the clearance of Aβ.

### Additional animal models for studying AD-related complement activation

#### TAPP and 3xTg-AD mice

Mutations in the gene encoding for human tau protein have been linked to the development of frontotemporal dementia with parkinsonism [115]. By combining this mutation with the human APP and PS1 mutations associated with familial AD, animal models of AD have been produced in which NFTs as well as SPs are formed. Lewis et al. [116] crossed human APP_swe _mice (Tg2576) with mice expressing the transgene for a human tau mutation (JNPL3 mice) to generate a double mutant tau/APP mouse (the "TAPP mouse"). These mice develop SPs similar to APP mice (high numbers of plaques are present in older [8.5–15 months of age] mice, in the olfactory cortex, cingulate gyrus, amygdala, entorhinal cortex, and hippocampus), and older TAPP mice have NFTs, in association with increased astrocyte proliferation, in limbic areas. The plaques contain both Aβ_40 _and Aβ_42_. Oddo et al. [117] injected the human transgenes for APP and mutated tau into embryos of PS1 "knock-in" mice, generating the "3xTg-AD" mouse which develops both SPs and NFTs in an age-related, region-specific manner. Aβ deposition in these animals precedes NFT formation, with extracellular Aβ (primarily Aβ_42_) detected in the frontal cortex by 6 months of age, and in other cortical regions and hippocampus by 12 months. Many of the extracellular Aβ deposits are thioflavin-S-positive and are associated with reactive astrocytes. Phosphorylated tau initially appears in the hippocampus and subsequently in cortical regions; it is detected within neurons by 12–15 months and within dystrophic neurites at 18 months. Though Aβ immunoreactivity precedes that of tau, these proteins co-localize to the same neurons. The presence of NFTs as well as SPs suggests that the 3xTg-AD and TAPP models may be more relevant than APP or APP/PS-1 mice for studying the significance of complement activation in the development of AD-type pathology. Potential drawbacks for using these models for complement-related studies include, as discussed earlier, functional deficiencies in activation of mouse complement [93], decreased complement levels in common laboratory mouse strains [94], and the decreased efficiency of binding of mouse C1q by the human Aβ within the SPs in these animals [95]. It is not known whether a similar decrease in the efficiency of activation of mouse complement occurs when mouse C1q binds to human, rather than murine, tau protein.

#### AD11 (anti-NGF) mouse

Ruberti et al. [118] developed a mouse transgenic model, the AD11 mouse, in which neutralizing antibody to nerve growth factor (NGF) is secreted by neurons and glial cells. NGF exerts trophic effects on basal forebrain cholinergic neurons and is widely distributed in these neurons [119]; the local secretion of anti-NGF antibody in these mice results in marked loss of basal forebrain cholinergic neurons. Aβ-containing plaques, tau hyperphosphorylation, and NFTs are present at 15–18 months of age. CNS production of anti-NGF antibody increases with age in these animals, therefore pathology develops only in adult mice. Extracellular deposition of APP is widespread in the brain, including the cortex and hippocampus. Phosphorylated tau immunoreactivity is present in neurons and glia in the cortex and hippocampus, and intracellular NFTs, extracellular neurofibrillary deposits, neuropil threads, and dystrophic neurites are observed in the cortex. Behavioral abnormalities, including impaired object recognition and spatial learning, are associated with this neuropathology [120]. The Aβ-containing plaques in the AD11 mouse are of murine, rather than human, origin, allowing the problem of the poor efficiency of activation of mouse complement by human Aβ [95] to be overcome. However, it is unclear whether plaques in these animals contain Aβ in the β-pleated sheet conformation, which is thought to be the most effective conformation for activating complement [71]. The distribution of SPs and NFTs in this model is less similar to AD than for 3xTg-AD and TAPP mice, because in addition to the cortex and hippocampus, large numbers of APP-reactive structures are present in the neostriatum (where, in AD, plaques are primarily diffuse [121]), and in other areas of the brain. Despite these concerns, the AD11 mouse is attractive as a potential model for studying the significance of AD-related complement activation.

##### *Chlamydia pneumoniae*-infected mouse

*C. pneumoniae *is an intracellular, gram-negative or gram-variable bacterium long identified as a respiratory pathogen. It has more recently been demonstrated to be a causative agent in reactive arthritis [122] and to be associated with autoimmune disorders including multiple sclerosis [123] and atherosclerosis [124]. Some laboratories have also reported an association of this agent with AD [125-127], although this has not been confirmed by others [128-131]. A recent study by Little et al. [132] examined the hypothesis that experimental *C. pneumoniae *infection in BALB/c mice could produce AD-like pathology. Intranasal inoculation with *C. pneumoniae *resulted in deposition of Aβ_1–42 _in the hippocampus, amygdala, entorhinal cortex, perirhinal cortex, and thalamus by 3 months post-inoculation. The majority of these Aβ deposits appeared similar to diffuse plaques, though a small number of them were thioflavin-S-reactive. NFTs were not detected. The authors suggested that soluble factors such as lipopolysaccharides, which are present in the cell wall of all Chlamydiae [133], may have been responsible for the altered amyloid processing which resulted in Aβ deposition. Because the Aβ within the SPs in these animals is of endogenous origin, and because other chlamydial species have been shown to activate complement [134,135], the *C. pneumoniae*-infected mouse may offer a novel infectious model for studying the relationship of complement activation to the development of Aβ-containing plaques.

#### Aged dogs

Old dogs, in particular the beagle, have been extensively investigated as a model for CNS Aβ deposition and associated age-related cognitive dysfunction. Aβ deposits are detectable in the brains of most older dogs [136]. The regional distribution of Aβ in the dog brain resembles that in humans, found initially in the prefrontal cortex, subsequently in entorhinal and parietal cortices, and lastly in occipital cortex [137]. Aβ_42 _is the predominant type of Aβ deposited in plaques [138]. Canine plaques are nonfibrillar and do not contain neuritic elements; thus, they resemble diffuse Aβ deposits in the human brain, but not the mature plaques predominating in AD. The neuropathological findings in old dogs also differ from AD in that activated glial cells are rarely associated with Aβ deposits, and NFTs are not detected [136,139]. Age-related cognitive impairment, termed "canine cognitive dysfunction syndrome," occurs in some older dogs and correlates with Aβ deposition in the hippocampus and frontal cortex [140,141]. The endogenous nature of the deposited Aβ in old dog brain, and similarities between canine and human Aβ in their patterns of regional deposition, suggest that this model may be useful for studying the relationship between complement activation and plaque formation.

#### Non-human primates

Age-related formation of SPs has been reported in a variety of non-human primates including the cynomolgus monkey [142], rhesus monkey [143], chimpanzee [144], and marmoset [145]. Aβ within these plaques is predominantly Aβ_40 _[146]. NFTs apparently do not form in the brains of most aged primates, with a few exceptions. The brain of the aged baboon contains phosphorylated tau protein [147,148], and an age-related accumulation of tau also occurs in the neocortex of the mouse lemur [149-151]. In this latter species, Aβ deposition occurs in the cerebral cortex and amygdala but is not age-dependent [151]. The mouse lemur appears to be the most promising primate species to date for studying the significance of AD-related complement activation because of the presence of NFTs as well as plaques.

#### Other animal species

Scattered reports of AD-type pathology in other species have also appeared. Adding trace amounts of copper to the water supply of cholesterol-fed rabbits results in Aβ deposition within SP-like structures in the hippocampus and temporal cortex, with associated learning deficits [152]. The neuropathology in the aged cat is similar to that in the old dog in that Aβ is deposited only as diffuse, Aβ_42_-containing plaques, and NFTs are not detected [138]. A report of AD-type pathology in an aged wolverine [153] described neuritic as well as diffuse plaques in the cortex and hippocampus, and intracellular NFTs containing phosphorylated tau protein in cortical and hippocampal neurons. Finally, the aged polar bear brain also contains both diffuse plaques and NFTs [154]. While the neuropathological findings in the aged wolverine and polar bear resemble AD more closely than in most species examined to date, their inaccessibility to laboratory researchers limits the usefulness of these species for studies of AD-related complement activation.

## Conclusions

1. Complement activation has been extensively studied in the AD brain. There is convincing evidence for activation of both the classical and alternative pathways, resulting in full activation as indicated by the presence of the MAC. Both aggregated Aβ (in SPs) and phosphorylated tau (in NFTs) are likely to be responsible for this activation.

2. Because complement activation generates both both neuroprotective and neurotoxic effects, the significance of increased complement activation in the development and progression of AD is unclear.

3. An optimal animal model for studying the significance of complement activation in the development of AD-type pathology would have complete activation of this process, with co-localization of complement activation proteins with SPs and with NFTs (if present). Other desirable features include early complement activation prior to the development of extensive neuropathology, increased CNS production of native complement proteins, and both classical and alternative pathway activation.

4. Surprisingly little is known about the extent of complement activation in animal models of AD. The postischemic hyperthermic rat [103] is the only animal model of AD in which full complement activation has been reported. The few studies with APP-transgenic mice have yielded conflicting results, with one investigation suggesting a neuroprotective role for complement activation [88], while another found that early complement activation (as indicated by C1q deposition) was associated with a loss of neuronal integrity [99]. Transgenic mouse models may be problematic for studies of AD-related complement activation because of inherent deficiencies in mouse complement activation and inefficient activation of mouse complement by the human Aβ present in the SPs in these animals. Other animal models in which SPs (and NFTs, if present) are of endogenous, rather than human, origin offer alternatives to transgenic mice for studying this issue.

5. The extent of complement activation and its association with neuropathology must be determined in animal models of AD to clarify the relevance of these models for investigating the significance of complement activation in the development of AD-type pathology.

## Abbreviations used

Aβ, amyloid beta; AD, Alzheimer's disease; APP, amyloid precursor protein; CNS, central nervous system; MAC, membrane attack complex; mRNA, messenger ribonucleic acid; NFTs, neurofibrillary tangles; NGF, nerve growth factor; PS-1, presenilin-1; sCrry, soluble complement receptor-related protein y; SPs, senile plaque; TGF-β1, transforming growth factor beta1.

## Competing interests

The author declares that he has no competing interests.

## References

[B1] Bamberger ME, Landreth GE (2002). Inflammation, apoptosis, and Alzheimer's disease. Neuroscientist.

[B2] Gupta A, Pansari K (2003). Inflammation and Alzheimer's disease. Int J Clin Pract.

[B3] Hoozemans JJ, Veerhuis R, Rozemuller AJ, Eikelenboom P (2002). The pathological cascade of Alzheimer's disease: the role of inflammation and its therapeutic implications. Drugs Today (Barc).

[B4] McGeer EG, McGeer PL (2003). Inflammatory processes in Alzheimer's disease. Prog Neuropsychopharmacol Biol Psychiatry.

[B5] Akiyama H, Barger S, Barnum S, Bradt B, Bauer J, Cole GM, Cooper NR, Eikelenboom P, Emmerling M, Fiebich BL, Finch CE, Frautschy S, Griffin WS, Hampel H, Hull M, Landreth G, Lue L, Mrak R, Mackenzie IR, McGeer PL, O'Banion MK, Pachter J, Pasinetti G, Plata-Salaman C, Rogers J, Rydel R, Shen Y, Streit W, Strohmeyer R, Tooyoma I, Van Muiswinkel FL, Veerhuis R, Walker D, Webster S, Wegrzyniak B, Wenk G, Wyss-Coray T (2000). Inflammation and Alzheimer's disease. Neurobiol Aging.

[B6] Combs CK, Karlo JC, Kao SC, Landreth GE (2001). beta-Amyloid stimulation of microglia and monocytes results in TNFalpha-dependent expression of inducible nitric oxide synthase and neuronal apoptosis. J Neurosci.

[B7] Griffin WS, Mrak RE (2002). Interleukin-1 in the genesis and progression of and risk for development of neuronal degeneration in Alzheimer's disease. J Leukoc Biol.

[B8] Griffin WS, Sheng JG, Royston MC, Gentleman SM, McKenzie JE, Graham DI, Roberts GW, Mrak RE (1998). Glial-neuronal interactions in Alzheimer's disease: the potential role of a 'cytokine cycle' in disease progression. Brain Pathol.

[B9] McGeer PL, Schulzer M, McGeer EG (1996). Arthritis and anti-inflammatory agents as possible protective factors for Alzheimer's disease: a review of 17 epidemiologic studies. Neurology.

[B10] Benveniste EN, Nguyen VT, O'Keefe GM (2001). Immunological aspects of microglia: relevance to Alzheimer's disease. Neurochem Int.

[B11] Meda L, Baron P, Scarlato G (2001). Glial activation in Alzheimer's disease: the role of Abeta and its associated proteins. Neurobiol Aging.

[B12] Mrak RE, Griffin WS (2001). The role of activated astrocytes and of the neurotrophic cytokine S100B in the pathogenesis of Alzheimer's disease. Neurobiol Aging.

[B13] Emmerling MR, Watson MD, Raby CA, Spiegel K (2000). The role of complement in Alzheimer's disease pathology. Biochim Biophys Acta.

[B14] McGeer PL, McGeer EG (2002). The possible role of complement activation in Alzheimer disease. Trends Mol Med.

[B15] Tenner AJ (2001). Complement in Alzheimer's disease: opportunities for modulating protective and pathogenic events. Neurobiol Aging.

[B16] Abraham CR (2001). Reactive astrocytes and alpha1-antichymotrypsin in Alzheimer's disease. Neurobiol Aging.

[B17] Kovacs DM (2000). alpha2-macroglobulin in late-onset Alzheimer's disease. Exp Gerontol.

[B18] Loeffler DA, Sima AA, LeWitt PA (2001). Ceruloplasmin immunoreactivity in neurodegenerative disorders. Free Radic Res.

[B19] Wood JA, Wood PL, Ryan R, Graff-Radford NR, Pilapil C, Robitaille Y, Quirion R (1993). Cytokine indices in Alzheimer's temporal cortex: no changes in mature IL-1 beta or IL-1RA but increases in the associated acute phase proteins IL-6, alpha 2-macroglobulin and C-reactive protein. Brain Res.

[B20] McGeer PL, Rogers J (1992). Anti-inflammatory agents as a therapeutic approach to Alzheimer's disease. Neurology.

[B21] Rogers J, Kirby LC, Hempelman SR, Berry DL, McGeer PL, Kaszniak AW, Zalinski J, Cofield M, Mansukhani L, Willson P, Kogan F (1993). Clinical trial of indomethacin in Alzheimer's disease. Neurology.

[B22] Aisen PS, Davis KL, Berg JD, Schafer K, Campbell K, Thomas RG, Weiner MF, Farlow MR, Sano M, Grundman M, Thal LJ (2000). A randomized controlled trial of prednisone in Alzheimer's disease. Alzheimer's Disease Cooperative Study. Neurology.

[B23] Aisen PS, Schafer KA, Grundman M, Pfeiffer E, Sano M, Davis KL, Farlow MR, Jin S, Thomas RG, Thal LJ (2003). Alzheimer's Disease Cooperative Study. Effects of rofecoxib or naproxen vs placebo on Alzheimer disease progression: a randomized controlled trial. JAMA.

[B24] Sainetti SM, Ingram DM, Talwalker S, Geis GS (2000). Results of a double-blind, randomized, placebo-controlled study of celecoxib in the treatment of progression of Alzheimer's disease [abstract]. Sixth International Stockholm/Springfield Symposium on Advances in Alzheimer Therapy.

[B25] Van Gool WA, Weinstein HC, Scheltens P, Walstra GJ, Scheltens PK (2001). Effect of hydroxychloroquine on progression of dementia in early Alzheimer's disease: an 18-month randomised, double-blind, placebo-controlled study. Lancet.

[B26] Shen Y, Meri S (2003). Yin and Yang: complement activation and regulation in Alzheimer's disease. Prog Neurobiol.

[B27] Wyss-Coray T, Mucke L (2002). Inflammation in neurodegenerative disease–a double-edged sword. Neuron.

[B28] Neumann H (2000). The immunological microenvironment in the CNS: implications on neuronal cell death and survival. J Neural Transm Suppl.

[B29] Polazzi E, Contestabile A (2002). Reciprocal interactions between microglia and neurons: from survival to neuropathology. Rev Neurosci.

[B30] van Beek J, Elward K, Gasque P (2003). Activation of complement in the central nervous system: roles in neurodegeneration and neuroprotection. Ann N Y Acad Sci.

[B31] Barnum SR (1995). Complement biosynthesis in the central nervous system. Crit Rev Oral Biol Med.

[B32] Eikelenboom P, Hack CE, Rozemuller JM, Stam FC (1989). Complement activation in amyloid plaques in Alzheimer's dementia. Virchows Arch B Cell Pathol Incl Mol Pathol.

[B33] Eikelenboom P, Stam FC (1982). Immunoglobulins and complement factors in senile plaques. An immunoperoxidase study. Acta Neuropathol (Berl).

[B34] Eikelenboom P, Stam FC (1984). An immunohistochemical study on cerebral vascular and senile plaque amyloid in Alzheimer's dementia. Virchows Arch B Cell Pathol Incl Mol Pathol.

[B35] Ishii T, Haga S (1984). Immuno-electron-microscopic localization of complements in amyloid fibrils of senile plaques. Acta Neuropathol (Berl).

[B36] Veerhuis R, Janssen I, Hack CE, Eikelenboom P (1996). Early complement components in Alzheimer's disease brains. Acta Neuropathol (Berl).

[B37] Veerhuis R, van der Valk P, Janssen I, Zhan SS, Van Nostrand WE, Eikelenboom P (1995). Complement activation in amyloid plaques in Alzheimer's disease brains does not proceed further than C3. Virchows Arch.

[B38] Itagaki S, Akiyama H, Saito H, McGeer PL (1994). Ultrastructural localization of complement membrane attack complex (MAC)-like immunoreactivity in brains of patients with Alzheimer's disease. Brain Res.

[B39] Lue LF, Brachova L, Civin WH, Rogers J (1996). Inflammation, A beta deposition, and neurofibrillary tangle formation as correlates of Alzheimer's disease neurodegeneration. J Neuropathol Exp Neurol.

[B40] McGeer PL, Akiyama H, Itagaki S, McGeer EG (1989). Activation of the classical complement pathway in brain tissue of Alzheimer patients. Neurosci Lett.

[B41] McGeer PL, Walker DG, Akiyama H, Kawamata T, Guan AL, Parker CJ, Okada N, McGeer EG (1991). Detection of the membrane inhibitor of reactive lysis (CD59) in diseased neurons of Alzheimer brain. Brain Res.

[B42] Webster S, Lue L-F, Brachova L, Tenner AJ, McGeer PL, Terai K, Walker DG, Bradt B, Cooper NR, Rogers J (1997). Molecular and cellular characterization of the membrane attack complex, C5b-9, in Alzheimer's disease. Neurobiol Aging.

[B43] Webster S, Glabe C, Rogers J (1995). Multivalent binding of complement protein C1q to the amyloid beta-peptide (A beta) promotes the nucleation phase of A beta aggregation. Biochem Biophys Res Commun.

[B44] Webster S, O'Barr S, Rogers J (1994). Enhanced aggregation and beta structure of amyloid beta peptide after coincubation with C1q. J Neurosci Res.

[B45] Schultz J, Schaller J, McKinley M, Bradt B, Cooper N, May P, Rogers J (1994). Enhanced cytotoxicity of amyloid beta-peptide by a complement dependent mechanism. Neurosci Lett.

[B46] Nolte C, Moller T, Walter T, Kettenmann H (1996). Complement 5a controls motility of murine microglial cells in vitro via activation of an inhibitory G-protein and the rearrangement of the actin cytoskeleton. Neuroscience.

[B47] Yao J, Harvath L, Gilert DL, Colton CA (1990). Chemotaxis by a CNS macrophage, the microglia. J Neurosci Res.

[B48] O'Barr S, Cooper NR (2000). The C5a complement activation peptide increases IL-1beta and IL-6 release from amyloid-beta primed human monocytes: implications for Alzheimer's disease. J Neuroimmunol.

[B49] Veerhuis R, Van Breemen MJ, Hoozemans JM, Morbin M, Ouladhadj J, Tagliavini F, Eikelenboom P (2003). Amyloid beta plaque-associated proteins C1q and SAP enhance the Abeta1–42 peptide-induced cytokine secretion by adult human microglia in vitro. Acta Neuropathol (Berl).

[B50] Shen Y, Halperin JA, Lee CM (1995). Complement-mediated neurotoxicity is regulated by homologous restriction. Brain Res.

[B51] Osaka H, Mukherjee P, Aisen PS, Pasinetti GM (1999). Complement-derived anaphylatoxin C5a protects against glutamate-mediated neurotoxicity. J Cell Biochem.

[B52] van Beek J, Nicole O, Ali C, Ischenko A, MacKenzie ET, Buisson A, Fontaine M (2001). Complement anaphylatoxin C3a is selectively protective against NMDA-induced neuronal cell death. Neuroreport.

[B53] O'Barr SA, Caguioa J, Gruol D, Perkins G, Ember JA, Hugli T, Cooper NR (2001). Neuronal expression of a functional receptor for the C5a complement activation fragment. J Immunol.

[B54] Mukherjee P, Pasinetti GM (2001). Complement anaphylatoxin C5a neuroprotects through mitogen-activated protein kinase-dependent inhibition of caspase 3. J Neurochem.

[B55] Soane L, Cho HJ, Niculescu F, Rus H, Shin ML (2001). C5b-9 terminal complement complex protects oligodendrocytes from death by regulating Bad through phosphatidylinositol 3-kinase/Akt pathway. J Immunol.

[B56] Frautschy SA, Hammer H, Hu S, Hu W, Oh M, Miller SA, Lim GP, Harris-White ME, Tenner AJ (2003). C1q stimulates Abeta clearance but worsens memory in Alzheimer model: too much C1q may be worse than too little. Program No 66712 Abstract Viewer/Itinerary Planner.

[B57] Webster SD, Yang AJ, Margol L, Garzon-Rodriguez W, Glabe CG, Tenner AJ (2000). Complement component C1q modulates the phagocytosis of Abeta by microglia. Exp Neurol.

[B58] Sahu A, Lambris JD (2000). Complement inhibitors: a resurgent concept in anti-inflammatory therapeutics. Immunopharmacol.

[B59] Morgan BP, Harris CL (2003). Complement therapeutics; history and current progress. Mol Immunol.

[B60] De Simoni MG, Storini C, Barba M, Catapano L, Arabia AM, Rossi E, Bergamaschini L (2003). Neuroprotection by complement (C1) inhibitor in mouse transient brain ischemia. J Cereb Blood Flow Metab.

[B61] Kirschfink M (2002). C1-inhibitor and transplantation. Immunobiology.

[B62] Quigg RJ (2003). Role of complement and complement regulatory proteins in glomerulonephritis. Springer Semin Immunopathol.

[B63] de Zwaan C, van Dieijen-Visser MP, Hermens WT (2003). Prevention of cardiac cell injury during acute myocardial infarction: possible role for complement inhibition. Am J Cardiovasc Drugs.

[B64] Farkas H, Harmat G, Fust G, Varga L, Visy B (2002). Clinical management of hereditary angio-oedema in children. Pediatr Allergy Immunol.

[B65] Rozemuller AJ, Eikelenboom P, Theeuwes JW, Jansen Steur EN, de Vos RA (2000). Activated microglial cells and complement factors are unrelated to cortical Lewy bodies. Acta Neuropathol (Berl).

[B66] Stoltzner SE, Grenfell TJ, Mori C, Wisniewski KE, Wisniewski TM, Selkoe DJ, Lemere CA (2000). Temporal accrual of complement proteins in amyloid plaques in Down's syndrome with Alzheimer's disease. Am J Pathol.

[B67] Rostagno A, Revesz T, Lashley T, Tomidokoro Y, Magnotti L, Braendgaard H, Plant G, Bojsen-Moller M, Holton J, Frangione B, Ghiso J (2002). Complement activation in chromosome 13 dementias. Similarities with Alzheimer's disease. J Biol Chem.

[B68] Bradt BM, Kolb WP, Cooper NR (1998). Complement-dependent proinflammatory properties of the Alzheimer's disease beta-peptide. J Exp Med.

[B69] Rogers J, Cooper NR, Webster S, Schultz J, McGeer PL, Styren SD, Civin WH, Brachova L, Bradt B, Ward P, Lieberburg I (1992). Complement activation by β-amyloid in Alzheimer disease. Proc Natl Acad Sci USA.

[B70] Shen Y, Lue L, Yang L, Roher A, Kuo Y, Strohmeyer R, Goux WJ, Lee V, Johnson GV, Webster SD, Cooper NR, Bradt B, Rogers J (2001). Complement activation by neurofibrillary tangles in Alzheimer's disease. Neurosci Lett.

[B71] Webster S, Bradt B, Rogers J, Cooper N (1997). Aggregation state-dependent activation of the classical complement pathway by the amyloid beta peptide. J Neurochem.

[B72] Bergamaschini L, Canziani S, Bottasso B, Cugno M, Braidotti P, Agostoni A (1999). Alzheimer's beta-amyloid peptides can activate the early components of complement classical pathway in a C1q-independent manner. Clin Exp Immunol.

[B73] Akiyama H, Mori H, Saido T, Kondo H, Ikeda K, McGeer PL (1999). Occurrence of the diffuse amyloid beta-protein (Abeta) deposits with numerous Abeta-containing glial cells in the cerebral cortex of patients with Alzheimer's disease. Glia.

[B74] Rozemuller JM, van der Valk P, Eikelenboom P (1992). Activated microglia and cerebral amyloid deposits in Alzheimer's disease. Res Immunol.

[B75] Lue LH, Rogers J (1992). Full complement activation fails in diffuse plaques of the Alzheimer's disease cerebellum. Dementia.

[B76] Fonseca MI, Kawas CH, Troncoso JC, Tenner AJ (2004). Neuronal localization of C1q in preclinical Alzheimer's disease. Neurobiol Dis.

[B77] Loeffler DA, Camp DM, Schonberger M, Singer DJ, LeWitt PA (2004). ELISA measurement of C4d and iC3b in Alzheimer's disease and normal brain specimens. Neurobiol Aging.

[B78] Shen Y, Li R, McGeer EG, McGeer PL (1997). Neuronal expression of mRNAs for complement proteins of the classical pathway in Alzheimer brain. Brain Res.

[B79] Walker DG, McGeer PL (1992). Complement gene expression in human brain: comparison between normal and Alzheimer disease cases. Brain Res Mol Brain Res.

[B80] Yasojima K, Schwab C, McGeer EG, McGeer PL (1999). Up-regulated production and activation of the complement system in Alzheimer's disease brain. Am J Pathol.

[B81] Walker DG, Yasuhara O, Patston PA, McGeer EG, McGeer PL (1995). Complement C1 inhibitor is produced by brain tissue and is cleaved in Alzheimer disease. Brain Res.

[B82] Yasojima K, McGeer EG, McGeer PL (1999). Complement regulators C1 inhibitor and CD59 do not significantly inhibit complement activation in Alzheimer disease. Brain Res.

[B83] Yang L-B, Li R, Meri S, Rogers J, Shen Y (2000). Deficiency of complement defense protein CD59 may contribute to neurodegeneration in Alzheimer's disease. J Neurosci.

[B84] Strohmeyer R, Ramirez M, Cole GJ, Mueller K, Rogers J (2002). Association of factor H of the alternative pathway of complement with agrin and complement receptor 3 in the Alzheimer's disease brain. J Neuroimmunol.

[B85] Strohmeyer R, Shen Y, Rogers J (2000). Detection of complement alternative pathway mRNA and proteins in the Alzheimer's disease brain. Brain Res Mol Brain Res.

[B86] Janus C, Phinney AL, Chishti MA, Westaway D (2001). New developments in animal models of Alzheimer's disease. Curr Neurol Neurosci Rep.

[B87] Wyss-Coray T, Lin C, Yan F, Yu GQ, Rohde M, McConlogue L, Masliah E, Mucke L (2001). TGF-beta1 promotes microglial amyloid-beta clearance and reduces plaque burden in transgenic mice. Nat Med.

[B88] Wyss-Coray T, Yan F, Lin AH, Lambris JD, Alexander JJ, Quigg RJ, Masliah E (2002). Prominent neurodegeneration and increased plaque formation in complement-inhibited Alzheimer's mice. Proc Natl Acad Sci USA.

[B89] Lindorfer MA, Hahn CS, Foley PL, Taylor RP (2001). Heteropolymer-mediated clearance of immune complexes via erythrocyte CR1: mechanisms and applications. Immunol Rev.

[B90] Molina H, Wong W, Kinoshita T, Brenner C, Foley S, Holers VM (1992). Distinct receptor and regulatory properties of recombinant mouse complement receptor 1 (CR1) and Crry, the two genetic homologues of human CR1. J Exp Med.

[B91] Yu JX, Bradt BM, Hsiao K, Carrol MC, Cooper NR (2000). Amyloid plaques in a transgenic mouse model of Alzheimer's disease contain complement components and pro-inflammatory cytokines. Program No 4911 2000 Abstract Viewer/Itinerary Planner.

[B92] McGeer PL, Schwab C, Staufenbiel M, Hosokawa M, McGeer EG (2002). Amyloid transgenic mice: an incomplete model of Alzheimer disease. Program No 2952 2002 Abstract Viewer/Itinerary Planner.

[B93] Ebanks RO, Isenman DE (1996). Mouse complement component C4 is devoid of classical pathway C5 convertase subunit activity. Mol Immunol.

[B94] Ong GL, Mattes MJ (1989). Mouse strains with typical mammalian levels of complement activity. J Immunol Methods.

[B95] Webster SD, Tenner AJ, Poulos TL, Cribbs DH (1999). The mouse C1q A-chain sequence alters beta-amyloid-induced complement activation. Neurobiol Aging.

[B96] Cruts M, Van Broeckhoven C (1998). Presenilin mutations in Alzheimer's disease. Hum Mutat.

[B97] Holcomb L, Gordon MN, McGowan E, Yu X, Benkovic S, Jantzen P, Wright K, Saad I, Mueller R, Morgan D, Sanders S, Zehr C, O'Campo K, Hardy J, Prada CM, Eckman C, Younkin S, Hsiao K, Duff K (1998). Accelerated Alzheimer-type phenotype in transgenic mice carrying both mutant amyloid precursor protein and presenilin 1 transgenes. Nat Med.

[B98] Matsuoka Y, Picciano M, Malester B, LaFrancois J, Zehr C, Daeschner JM, Olschowka JA, Fonseca MI, O'Banion MK, Tenner AJ, Lemere CA, Duff K (2001). Inflammatory responses to amyloidosis in a transgenic mouse model of Alzheimer's disease. Am J Pathol.

[B99] Fonseca MI, Zhou J, Botto M, Tenner AJ (2004). Absence of C1q leads to less neuropathology in transgenic mouse models of Alzheimer's disease. J Neurosci.

[B100] Hsiao K, Chapman P, Nilsen S, Eckman C, Harigaya Y, Younkin S, Yang F, Cole G (1996). Correlative memory deficits, Abeta elevation, and amyloid plaques in transgenic mice. Science.

[B101] Botto M, Dell'Agnola C, Bygrave AE, Thompson EM, Cook HT, Petry F, Loos M, Pandolfi PP, Walport MJ (1998). Homozygous C1q deficiency causes glomerulonephritis associated with multiple apoptotic bodies. Nat Genet.

[B102] Coimbra C, Drake M, Boris-Moller F, Wieloch T (1996). Long-lasting neuroprotective effect of postischemic hypothermia and treatment with an anti-inflammatory/antipyretic drug. Evidence for chronic encephalopathic processes following ischemia. Stroke.

[B103] Sinigaglia-Coimbra R, Cavalheiro EA, Coimbra CG (2002). Postischemic hyperthermia induces Alzheimer-like pathology in the rat brain. Acta Neuropathol (Berl).

[B104] Lee JH, Kim SR, Bae CS, Kim D, Hong H, Nah S (2002). Protective effect of ginsenosides, active ingredients of Panax ginseng, on kainic acid-induced neurotoxicity in rat hippocampus. Neurosci Lett.

[B105] Hyman BT, Van Horsen GW, Damasio AR, Barnes CL (1984). Alzheimer's disease: cell-specific pathology isolates the hippocampal formation. Science.

[B106] Johnson SA, Lampert-Etchells M, Pasinetti GM, Rozovsky I, Finch CE (1992). Complement mRNA in the mammalian brain: responses to Alzheimer's disease and experimental brain lesioning. Neurobiol Aging.

[B107] Johnson S, Young-Chan CS, Laping NJ, Finch CE (1996). Perforant path transection induces complement C9 deposition in hippocampus. Exp Neurol.

[B108] Pasinetti GM, Johnson SA, Rozovsky I, Lampert-Etchells M, Morgan DG, Gordon MN, Morgan TE, Willoughby D, Finch CE (1992). Complement C1qB and C4 mRNAs responses to lesioning in rat brain. Exp Neurol.

[B109] Rozovsky I, Morgan TE, Willoughby DA, Dugichi-Djordjevich MM, Pasinetti GM, Johnson SA, Finch CE (1994). Selective expression of clusterin (SGP-2) and complement C1qB and C4 during responses to neurotoxins in vivo and in vitro. Neuroscience.

[B110] Sahu A, Rawal N, Pangburn MK (1999). Inhibition of complement by covalent attachment of rosmarinic acid to activated C3b. Biochem Pharmacol.

[B111] Osakabe N, Yasuda A, Natsume M, Yoshikawa T (2004). Rosmarinic acid inhibits epidermal inflammatory responses: anticarcinogenic effect of *Perilla frutescens *extract in the murine two-stage skin model. Carcinogenesis.

[B112] Youn J, Lee KH, Won J, Huh SJ, Yun HS, Cho WG, Paik DJ (2003). Beneficial effects of rosmarinic acid on suppression of collagen induced arthritis. J Rheumatol.

[B113] Parejo I, Viladomat F, Bastida J, Schmeda-Hirschmann G, Burillo J, Codina C (2004). Bioguided isolation and identification of the nonvolatile antioxidant compounds from fennel (Foeniculum vulgare Mill.) waste. J Agric Food Chem.

[B114] Ono K, Hasegawa K, Naiki H, Yamada M (2004). Curcumin has potent anti-amyloidogenic effects for Alzheimer's beta-amyloid fibrils in vitro. J Neurosci Res.

[B115] Spillantini MG, Goedert M (1998). Tau protein pathology in neurodegenerative diseases. Trends Neurosci.

[B116] Lewis J, Dickson DW, Lin WL, Chisholm L, Corral A, Jones G, Yen SH, Sahara N, Skipper L, Yager D, Eckman C, Hardy J, Hutton M, McGowan E (2001). Enhanced neurofibrillary degeneration in transgenic mice expressing mutant tau and APP. Science.

[B117] Oddo S, Caccamo A, Shepherd JD, Murphy MP, Golde TE, Kayed R, Metherate R, Mattson MP, Akbari Y, LaFerla FM (2003). Triple-transgenic model of Alzheimer's disease with plaques and tangles: intracellular Abeta and synaptic dysfunction. Neuron.

[B118] Ruberti F, Capsoni S, Comparini A, Di Daniel E, Franzot J, Gonfloni S, Rossi G, Berardi N, Cattaneo A (2000). Phenotypic knockout of nerve growth factor in adult transgenic mice reveals severe deficits in basal forebrain cholinergic neurons, cell death in the spleen, and skeletal muscle dystrophy. J Neurosci.

[B119] Lauterborn JC, Isackson PJ, Gall CM (1991). Nerve growth factor mRNA-containing cells are distributed within regions of cholinergic neurons in the rat basal forebrain. J Comp Neurol.

[B120] Capsoni S, Ugolini G, Comparini A, Ruberti F, Berardi N, Cattaneo A (2000). Alzheimer-like neurodegeneration in aged antinerve growth factor transgenic mice. Proc Natl Acad Sci USA.

[B121] Gearing M, Levey AI, Mirra SS (1997). Diffuse plaques in the striatum in Alzheimer disease (AD): relationship to the striatal mosaic and selected neuropeptide markers. J Neuropathol Exp Neurol.

[B122] Braun J, Laitko S, Treharne J, Eggens U, Wu P, Distler A, Sieper J (1994). *Chlamydia pneumoniae *–a new causative agent of reactive arthritis and undifferentiated oligoarthritis. Ann Rheum Dis.

[B123] Sriram S, Stratton CW, Yao S, Tharp A, Ding L, Bannan JD, Mitchell WM (1999). *Chlamydia pneumoniae *infection of the central nervous system in multiple sclerosis. Ann Neurol.

[B124] Campbell LA, Kuo CC (2004). *Chlamydia pneumoniae *–an infectious risk factor for atherosclerosis?. Nat Rev Microbiol.

[B125] Balin BJ, Gerard HC, Arking EJ, Appelt DM, Branigan PJ, Abrams JT, Whittum-Hudson JA, Hudson AP (1998). Identification and localization of *Chlamydia pneumoniae *in the Alzheimer's brain. Med Microbiol Immunol (Berl).

[B126] Mahony JB, Woulfe J, Munoz D, Browning D, Chong S, Smieja M (2000). Identification of Chlamydiae pneumoniae in the Alzheimer's brain. World Alzheimer Congress.

[B127] Ossewaarde JM, Gielis-Proper SK, Meijer A, Roholl PJM, Saikku P Chlamydia pneumoniae antigens are present in the brains of Alzheimer patients, but not in the brains of patients with other dementias. In Proceedings of the 4th Meeting of European Society for Chlamydia Research: 20–23 August 2000; Helsinki, Finland.

[B128] Gieffers J, Reusche E, Solbach W, Maass M (2000). Failure to detect *Chlamydia pneumoniae *in brain sections of Alzheimer's disease patients. J Clin Microbiol.

[B129] Nochlin D, Shaw CM, Campbell LA, Kuo CC (1999). Failure to detect *Chlamydia pneumoniae *in brain tissues of Alzheimer's disease. Neurology.

[B130] Ring RH, Lyons JM (2000). Failure to detect *Chlamydia pneumoniae *in the late-onset Alzheimer's brain. J Clin Microbiol.

[B131] Wozniak MA, Cookson A, Wilcock GK, Itzhaki RF (2003). Absence of *Chlamydia pneumoniae *in brain of vascular dementia patients. Neurobiol Aging.

[B132] Little CS, Hammond CJ, MacIntyre A, Balin BJ, Appelt DM (2004). *Chlamydia pneumoniae *induces Alzheimer-like amyloid plaques in brains of BALB/c mice. Neurobiol Aging.

[B133] Jawetz E, Melnick JL, Adelberg EA (1972). Review of Medical Microbiology.

[B134] Hall RT, Strugnell T, Wu X, Devine DV, Stiver HG (1993). Characterization of kinetics and target proteins for binding of human complement component C3 to the surface-exposed outer membrane of *Chlamydia trachomatis *serovar L2. Infect Immun.

[B135] Megran DW, Stiver HG, Bowie WR (1985). Complement activation and stimulation of chemotaxis by *Chlamydia trachomatis.*. Infect Immun.

[B136] Satou T, Cummings BJ, Head E, Nielson KA, Hahn FF, Milgram NW, Velazquez P, Cribbs DH, Tenner AJ, Cotman CW (1997). The progression of beta-amyloid deposition in the frontal cortex of the aged canine. Brain Res.

[B137] Head E, McCleary R, Hahn FF, Milgram NW, Cotman CW (2000). Region-specific age at onset of beta-amyloid in dogs. Neurobiol Aging.

[B138] Cummings BJ, Satou T, Head E, Milgram NW, Cole GM, Savage MJ, Podlisny MB, Selkoe DJ, Siman R, Greenberg BD, Cotman CW (1996). Diffuse plaques contain C-terminal A beta 42 and not A beta 40: evidence from cats and dogs. Neurobiol Aging.

[B139] Cummings BJ, Su JH, Cotman CW, White R, Russell MJ (1993). Beta-amyloid accumulation in aged canine brain: a model of early plaque formation in Alzheimer's disease. Neurobiol Aging.

[B140] Cummings BJ, Head E, Afagh AJ, Milgram NW, Cotman CW (1996). Beta-amyloid accumulation correlates with cognitive dysfunction in the aged canine. Neurobiol Learn Mem.

[B141] Cummings BJ, Head E, Ruehl W, Milgram NW, Cotman CW (1996). The canine as an animal model of human aging and dementia. Neurobiol Aging.

[B142] Kimura N, Tanemura K, Nakamura S, Takashima A, Ono F, Sakakibara I, Ishii Y, Kyuwa S, Yoshikawa Y (2003). Age-related changes of Alzheimer's disease-associated proteins in cynomolgus monkey brains. Biochem Biophys Res Commun.

[B143] Sloane JA, Pietropaolo MF, Rosene DL, Moss MB, Peters A, Kemper T, Abraham CR (1997). Lack of correlation between plaque burden and cognition in the aged monkey. Acta Neuropathol (Berl).

[B144] Gearing M, Rebeck GW, Hyman BT, Tigges J, Mirra SS (1994). Neuropathology and apolipoprotein E profile of aged chimpanzees: implications for Alzheimer disease. Proc Natl Acad Sci USA.

[B145] Geula C, Nagykery N, Wu CK (2002). Amyloid-beta deposits in the cerebral cortex of the aged common marmoset (*Callithrix jacchus*): incidence and chemical composition. Acta Neuropathol (Berl).

[B146] Gearing M, Tigges J, Mori H, Mirra SS (1996). A beta40 is a major form of beta-amyloid in nonhuman primates. Neurobiol Aging.

[B147] Schultz C, Hubbard GB, Rub U, Braak E, Braak H (2000). Age-related progression of tau pathology in brains of baboons. Neurobiol Aging.

[B148] Schultz C, Hubbard GB, Tredici KD, Braak E, Braak H (2001). Tau pathology in neurons and glial cells of aged baboons. Adv Exp Med Biol.

[B149] Bons N, Jallageas V, Silhol S, Mestre-Frances N, Petter A, Delacourte A (1995). Immunocytochemical characterization of Tau proteins during cerebral aging of the lemurian primate *Microcebus murinus.*. C R Acad Sci III.

[B150] Bons N, Mestre N, Petter A (1992). Senile plaques and neurofibrillary changes in the brain of an aged lemurian primate, *Microcebus murinus.*. Neurobiol Aging.

[B151] Giannakopoulos P, Silhol S, Jallageas V, Mallet J, Bons N, Bouras C, Delaere P (1997). Quantitative analysis of tau protein-immunoreactive accumulations and beta amyloid protein deposits in the cerebral cortex of the mouse lemur, *Microcebus murinus*. Acta Neuropathol (Berl).

[B152] Sparks DL, Schreurs BG (2003). Trace amounts of copper in water induce beta-amyloid plaques and learning deficits in a rabbit model of Alzheimer's disease. Proc Natl Acad Sci USA.

[B153] Roertgen KE, Parisi JE, Clark HB, Barnes DL, O'Brien TD, Johnson KH (1996). Abeta-associated cerebral angiopathy and senile plaques with neurofibrillary tangles and cerebral hemorrhage in an aged wolverine (Gulo gulo). Neurobiol Aging.

[B154] Tekirian TL, Saido TC, Markesbery WR, Russell MJ, Wekstein DR, Patel E, Geddes JW (1998). N-terminal heterogeneity of parenchymal and cerebrovascular Abeta deposits. J Neuropathol Exp Neurol.

[B155] Bokisch VA, Muller-Eberhard  HJ, Cochrane CG (1969). Isolation of a fragment (C3a) of the  third component of human complement containing anaphylatoxin and chemotactic activity  and description of an anaphylatoxin inactivator of human serum.. J Exp Med.

[B156] Gorski JP, Hugli TE, Muller-Eberhard  HJ (1979). C4a: the third anaphylatoxin of the human  complement system.. Proc Natl Acad Sci U S A.

[B157] Gasque  P, Dean YD, McGreal EP, VanBeek J, Morgan BP (2000). Complement components of  the innate immune system in health and disease in the CNS.. Immunopharmacology.

